# Quantification of radial arterial pulse characteristics change during exercise and recovery

**DOI:** 10.1007/s12576-016-0515-7

**Published:** 2016-12-27

**Authors:** Anran Wang, Lin Yang, Weimin Wen, Song Zhang, Dongmei Hao, Syed G. Khalid, Dingchang Zheng

**Affiliations:** 1 College of Life Science and Bio-engineering, Beijing University of Technology, 100 Pingluoyuan Chaoyang District, Beijing, 100124 China; 2 Health and Wellbeing Academy, Faculty of Medical Science, Anglia Ruskin University, Chelmsford, CM1 1SQ UK

**Keywords:** Radial pulse, Exercise load, Pulse wave analysis, Blood pressure

## Abstract

It is physiologically important to understand the arterial pulse waveform characteristics change during exercise and recovery. However, there is a lack of a comprehensive investigation. This study aimed to provide scientific evidence on the arterial pulse characteristics change during exercise and recovery. Sixty-five healthy subjects were studied. The exercise loads were gradually increased from 0 to 125 W for female subjects and to 150 W for male subjects. Radial pulses were digitally recorded during exercise and 4-min recovery. Four parameters were extracted from the raw arterial pulse waveform, including the pulse amplitude, width, pulse peak and dicrotic notch time. Five parameters were extracted from the normalized radial pulse waveform, including the pulse peak and dicrotic notch position, pulse Area, Area_1_ and Area_2_ separated by notch point. With increasing loads during exercise, the raw pulse amplitude increased significantly with decreased pulse period, reduced peak and notch time. From the normalized pulses, the pulse Area, pulse Area_1_ and Area_2_ decreased, respectively, from 38 ± 4, 61 ± 5 and 23 ± 5 at rest to 34 ± 4, 52 ± 6 and 13 ± 5 at 150-W exercise load. During recovery, an opposite trend was observed. This study quantitatively demonstrated significant changes of radial pulse characteristics during different exercise loads and recovery phases.

## Introduction

Skeletal muscle contraction during exercise escalates oxygen consumption and the human body metabolism rate, leading to increased cardiac output. The increase in cardiac output during exercise is associated with the increase of heart rate and stroke volume, ultimately resulting in increased blood pressure.

Studying the cardiovascular function changes during exercise in comparison with resting condition is physiologically important [1]. Three main parameters including blood pressure, heart rate and blood flow are commonly used to assess the cardiovascular function [2–4]. Over many years, many researchers have focused on cardiovascular function change during exercise or recovery. The cardiopulmonary exercise test has been used to evaluate the cardiopulmonary function change by measuring heart rate, oxygen uptake, carbon dioxide discharge and minute ventilation in exercise [5]. The exercise cardiac contractility monitor developed by Xiao et al. has also been used to evaluate cardiac contractility capacity reserve by analyzing heart sound during exercise [6, 7]. The measurement accuracy was heavily affected by breathing, and it is not easy to operate. There were also studies investigating cardiovascular state changes during recovery phase, but they mainly focused on heart rate variability, not the cardiovascular parameters [8, 9].

Arterial pulse waveforms could be reliably and easily detected from the periphery. The characteristics of such waveforms contain information reflecting the cardiac function and peripheral resistance [10, 11]. It has been accepted that arterial pulse waves could be used to evaluate cardiovascular function and analyze cardiovascular hemodynamic characteristics in clinical medicine [12]. Some published studies used pulse wave velocity to assess the cardiovascular function of athletes [13, 14]. Munir et al. investigated the effect of exercise on the arterial pulse by analyzing pressure wave reflection and pulse wave velocity [15] and found that the time to the point of maximal diastolic augmentation was reduced during and immediately after exercise, which was similar to the baseline parameters at later stages in recovery. However, their research mainly focused on the pulse wave velocity. Many other waveform characteristics could be derived from the pulses. Furthermore, the effect of different exercise loads has not been comprehensively investigated. A comprehensive analysis of waveform characteristics change during exercise with different loads and during recovery is therefore necessary. This study aimed to provide scientific evidence on arterial pulse shape changes during exercise and recovery.

## Methods

### Subjects

Sixty-five nonsmoking healthy subjects (18 female and 47 male) were enrolled from the Beijing University of Technology. None of them were amateur or professional athletes. They had no history of cardiovascular diseases and were not under medication. The female subjects were not in a menstrual cycle phase. The study was fully approved by the local Ethics Committee, College of Life Science and Bio-engineering, Beijing University of Technology. The investigation conformed to the principles in the Declaration of Helsinki. All subjects gave written informed consent. The overall basic clinical information, including the age, height, weight and BMI, is shown in Table 1.

**Table 1:** Clinical variables from the 65 subjects studied

Variables	Male	Female	All
No.	47	18	65
Age, year	26 ± 3	22 ± 3	25 ± 3
Height, cm	174 ± 5	162 ± 5	170 ± 7
Weight, kg	67 ± 7	51 ± 6	62 ± 9
BMI, kg/m^2^	22 ± 2	19 ± 2	21 ± 2

*BMI* body mass index

### Experimental procedure

The experiment was performed in a quiet clinical measurement room. Subjects were asked to sit quietly for 10 min on a chair before the study. Baseline blood pressure and heart rate were measured before the exercise using a validated electronic sphygmomanometer (HEM-7124 from Omron Corp.). The pressure sensor was placed on the right wrist to record the arterial pulses using a PowerLab data collection system (ADInstruments Pty Ltd., PowerLab 8/35, Bella Vista NSW 2153, Australia) at a sampling rate of 1000 Hz. The sphygmomanometer cuff was left on the left arm during the experimental process.

Before the formal arterial pulse recording, subjects were asked to perform 30-s trail exercise on the cycle ergometer (Monark Pty Ltd., Ergomedic 839 E, Sweden) to ensure the pressure sensor was comfortably placed and to ensure good quality waveforms could be obtained. For the formal recording, a baseline recording of the radial pulse waveform was first recorded while the subject sat on the ergometer without any exercise load. Different exercise loads were then used, starting from 25 W to a maximum exercise load of 125 W for female subjects and to 150 W for male subjects. At each exercise load, 3 min of good quality of waveforms was recorded. There was a 1-min gap between each exercise load for automatic blood pressure measurements.

**Fig. 1:**
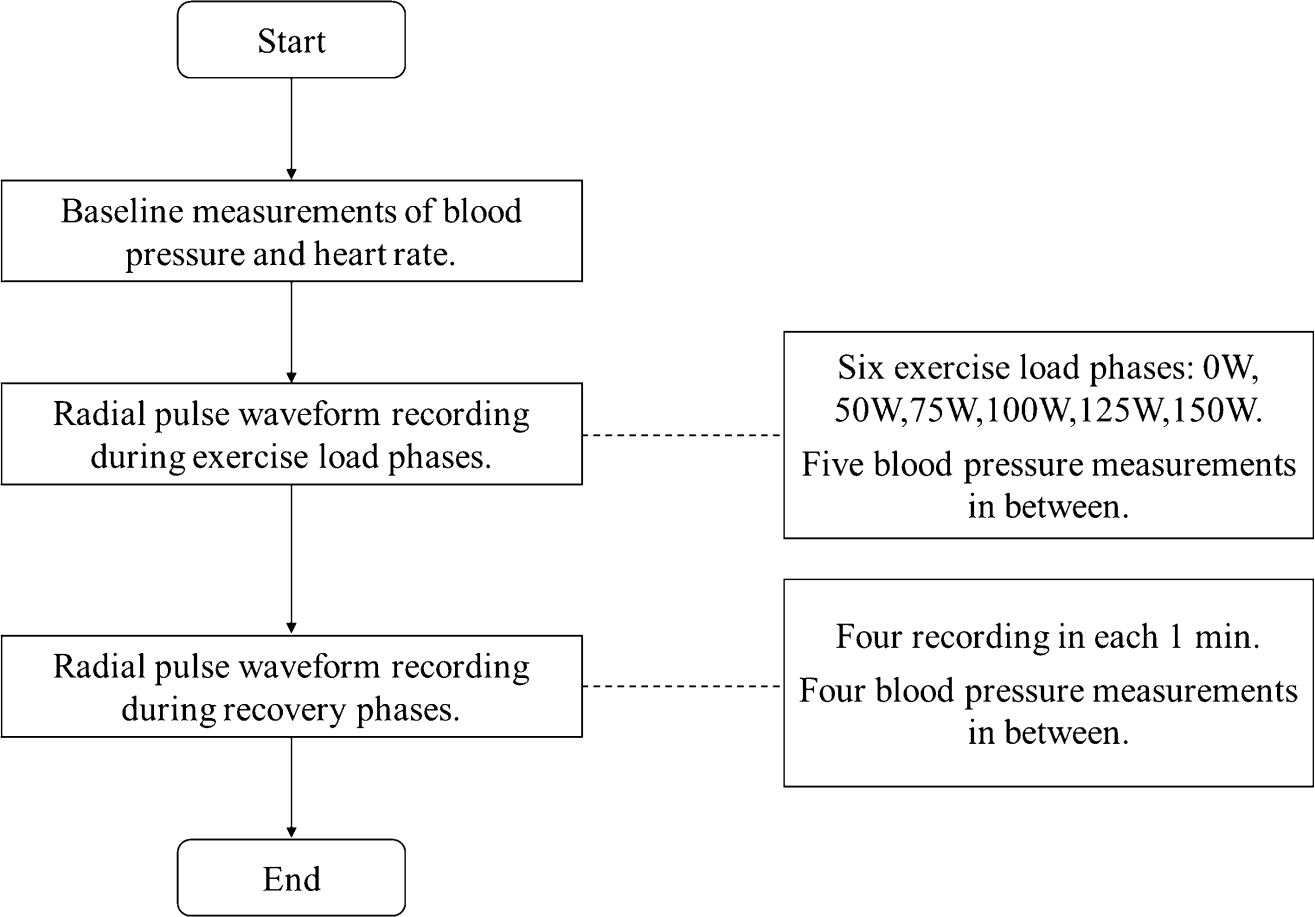
Experimental flow diagram

After the maximum load, each subject experienced a recovery period (at least 4 min). Four sections of radial pulse waveform recording (1 min for each session) were performed. There was a 1-min gap in between for automatic blood pressure measurement. The experimental procedure is shown in Fig. 1.

### Pulse characteristics determination

#### Pulse waveform pre-processing and arterial pulse normalization

**Fig. 2:**
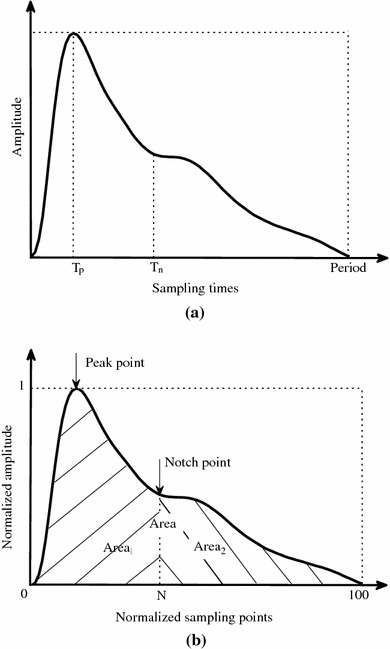
Illustration of the determination of waveform characteristics from the raw radial pulse waveform (**a**) and normalized radial pulse waveform (**b**). Four parameters were defined in the raw pulse waveform, including the pulse amplitude, pulse period, pulse peak time *T*_p_ and dicrotic notch time *T*_n_. **b** Five parameters were defined in the normalized pulse waveform, including the pulse peak point, pulse dicrotic notch point, pulse Area, pulse Area_1_ and pulse Area_2_

All the pulses from the last minute’s recording at each exercise load phase and the whole minute of recording at different recovery phases were used for off-line signal processing. The pulse waveform baseline drift was first removed [16]. All the pulses were then averaged to have a single reference pulse for different exercise loads and for the four recovery phases. Figure 2a shows one example of the averaged raw pulse with an exercise load of 0 W.

Next, all the pulses, separately for different exercise loads and for the four recovery phases, were normalized in both width (100 sampling points) and amplitude (0–1) from the foot of each pulse and then averaged to obtain a single reference normalized pulse for each subject, as shown in Fig. 2b.

#### Characteristic parameters from raw pulse waveforms

From the averaged raw waveform, four parameters were extracted as shown in Fig. 2a, including the pulse amplitude and width, pulse peak time (*T*_p_, determined from the first derivative radial pulse and corresponding to the first zero-crossing point after the pulse peak [17]) and dicrotic notch time (*T*_n_, determined from the second derivative radial pulse, corresponding to the maximum point after the pulse peak time *T*_p_ [17]).

#### Characteristic parameters from normalized pulse waveforms

From the normalized arterial pulse waveform, five parameters were extracted as shown in Fig. 2b, including the pulse peak position and dicrotic notch point (similar determination method as mentioned above) and total ‘Pulse Area’ under the waveform (which was computed from the normalized pulse waveform as: ${\text{Area}} = \int_{0}^{100} {Y (t ) {\text{d}}t}$ [18]).

‘Pulse Area_1_’ (which was computed from the normalized pulse waveform as ${\text{Area}}_{1} = \int_{0}^{N} {Y (t ) {\text{d}}t}$ [19]) and ‘Pulse Area_2_’ (${\text{Area}}_{2} = \int_{N}^{100} {Y (t ) {\text{d}}t}$ [19]).

### Statistical analyses

The mean ± SD of all the parameters (heart rate, blood pressure, pulse pressure; the pulse amplitude, pulse width, pulse peak time and pulse dicrotic notch time extracted from raw arterial waveform; the pulse peak position, pulse dicrotic notch position, pulse Area, and the pulse Area_1_ and pulse Area_2_ extracted from the normalized pulse) were calculated across all the subjects separately for different exercise loads and recovery phases. One-way analysis of variance was then performed using SPSS to compare whether there were significant differences between different exercise loads or recovery phases. An independent-samples *T* test was also performed to compare the difference of arterial pulse waveform characteristics between male and female subjects separately for different exercise loads and recovery phases. *P* < 0.05 was used as the significant criterion for all the parameters.

## Results

### Changes in physiological parameters during exercise and recovery

**Fig. 3:**
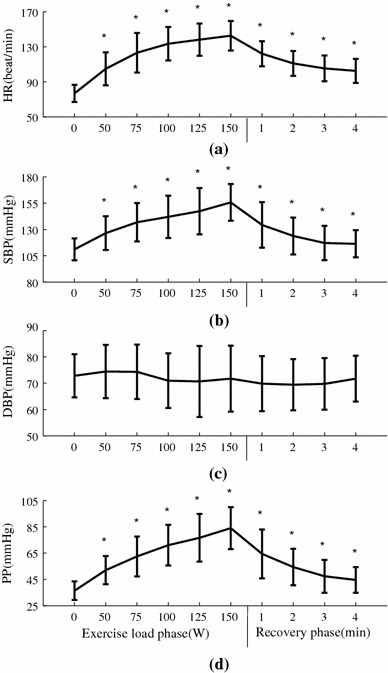
Physiological parameters (mean ± SD) at different exercise loads and during recovery from the 65 subjects studied. *P < 0.05 significantly different in comparison with the 0-W resting phase

As shown in Fig. 3, as expected, heart rate and blood pressure increased significantly with increasing exercise load (between baseline and maximum load; 77 ± 10 vs. 143 ± 17 bpm for HR, 111 ± 10 vs. 156 ± 18 mmHg for SBP and 36 ± 7 vs. 84 ± 16 mmHg for PP). After 4-min recovery, they decreased to 105 ± 15 bpm for HR, 117 ± 16 mmHg for SBP and 47 ± 12 mmHg for PP. In comparisons with resting phase, all the changes at the five exercise loads and during recovery were significant (all *P* < 0.05). It is also noted there were no significant differences in the DBP among all the exercise loads, recovery phase and the resting phase.

### Raw waveform characteristics changes during exercise and recovery

**Fig. 4:**
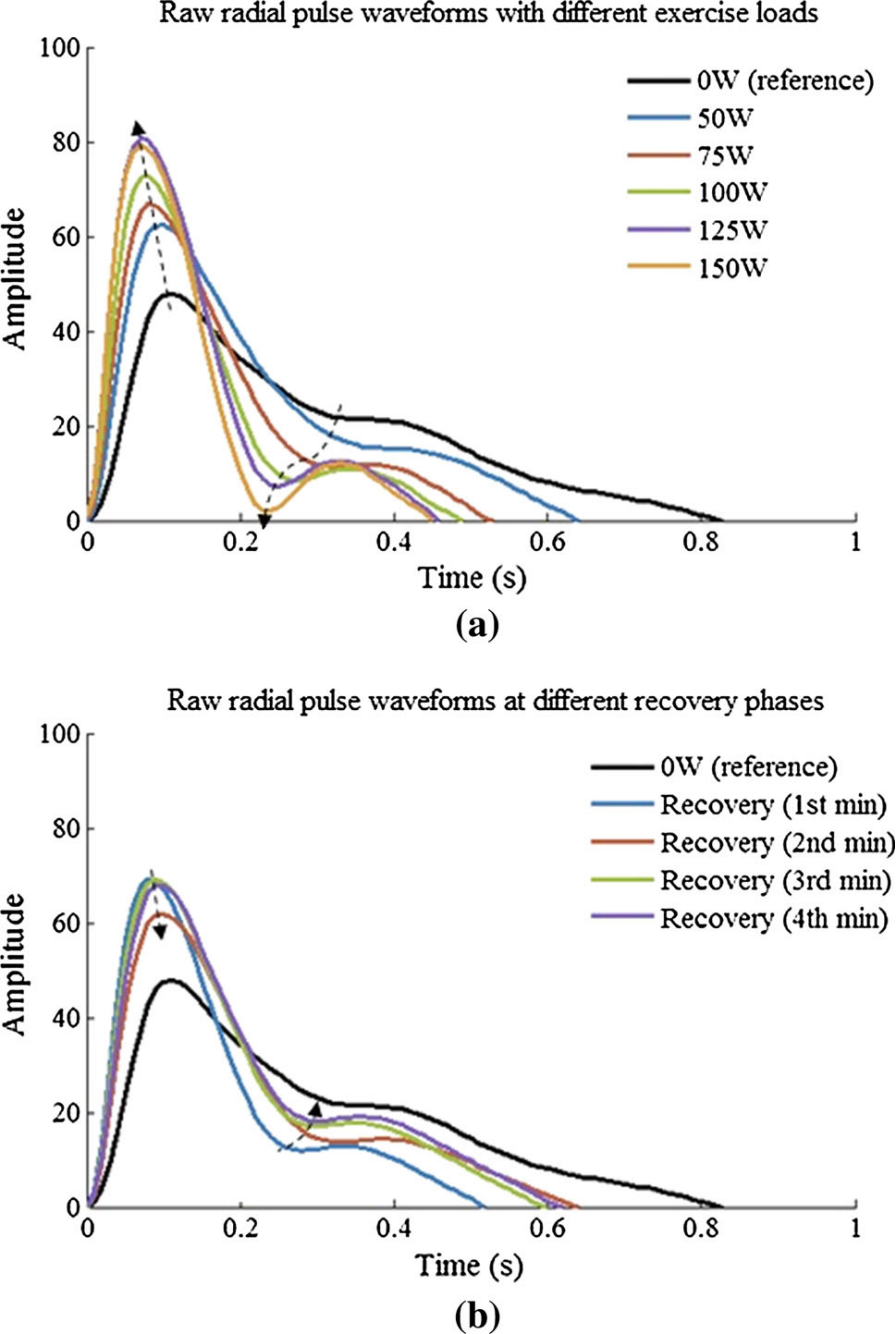
Raw radial pulse waveforms averaged from the 65 subjects studied. The *arrows* indicate the shift tendency of the peak points and notch points during exercise and recovery

**Fig. 5:**
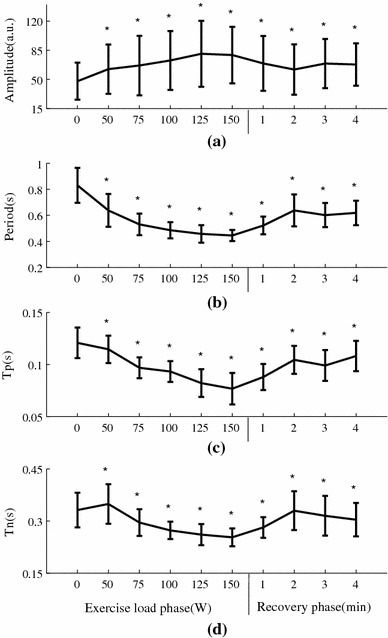
Raw radial pulse waveform parameters (mean ± SD) at different exercise loads and recovery phases from the 65 subjects studied. *P < 0.05 significantly different in comparison with the 0-W resting phase

Figure 4 shows the averaged raw radial pulse waveform of each phase from all the subjects, showing increased waveform amplitude and decreased waveform width with increasing exercise loads. During recovery, the amplitude decreased toward the baseline level and the pulse width increased. More specifically, as shown in Fig. 5, pulse amplitude (with arbitrary unit) increased from 48 ± 22 at rest to 62 ± 30, 73 ± 35 and 79 ± 34 at 50, 100 and 150 W and then decreased to 68 ± 25 at the end of the 4-min recovery phase. Correspondingly, pulse width increased from 0.83 ± 0.13 s at rest to 0.64 ± 0.13, 0.49 ± 0.06 and 0.45 ± 0.04 s at 50, 100 and 150 W and then decreased to 0.62 ± 0.09 s at the 4-min recovery phase. All the changes were significant (all *P* < 0.05).

In terms of peak time *T*_p_, it decreased significantly during exercise from 0.12 ± 0.01 s at rest to 0.11 ± 0.01, 0.09 ± 0.01 and 0.08 ± 0.02 s at 50, 100 and 150 W and then increased to 0.11 ± 0.01 s at the 4-min recovery phase. Correspondingly, the pulse notch time *T*_n_ decreased from 0.33 ± 0.05 s at rest to 0.25 ± 0.03 s at 150 W and then increased to 0.31 ± 0.05 s at the 4-min recovery phase. All the changes were significant (all *P* < 0.05).

### Normalized waveform characteristics changes during exercise and recovery

**Fig. 6:**
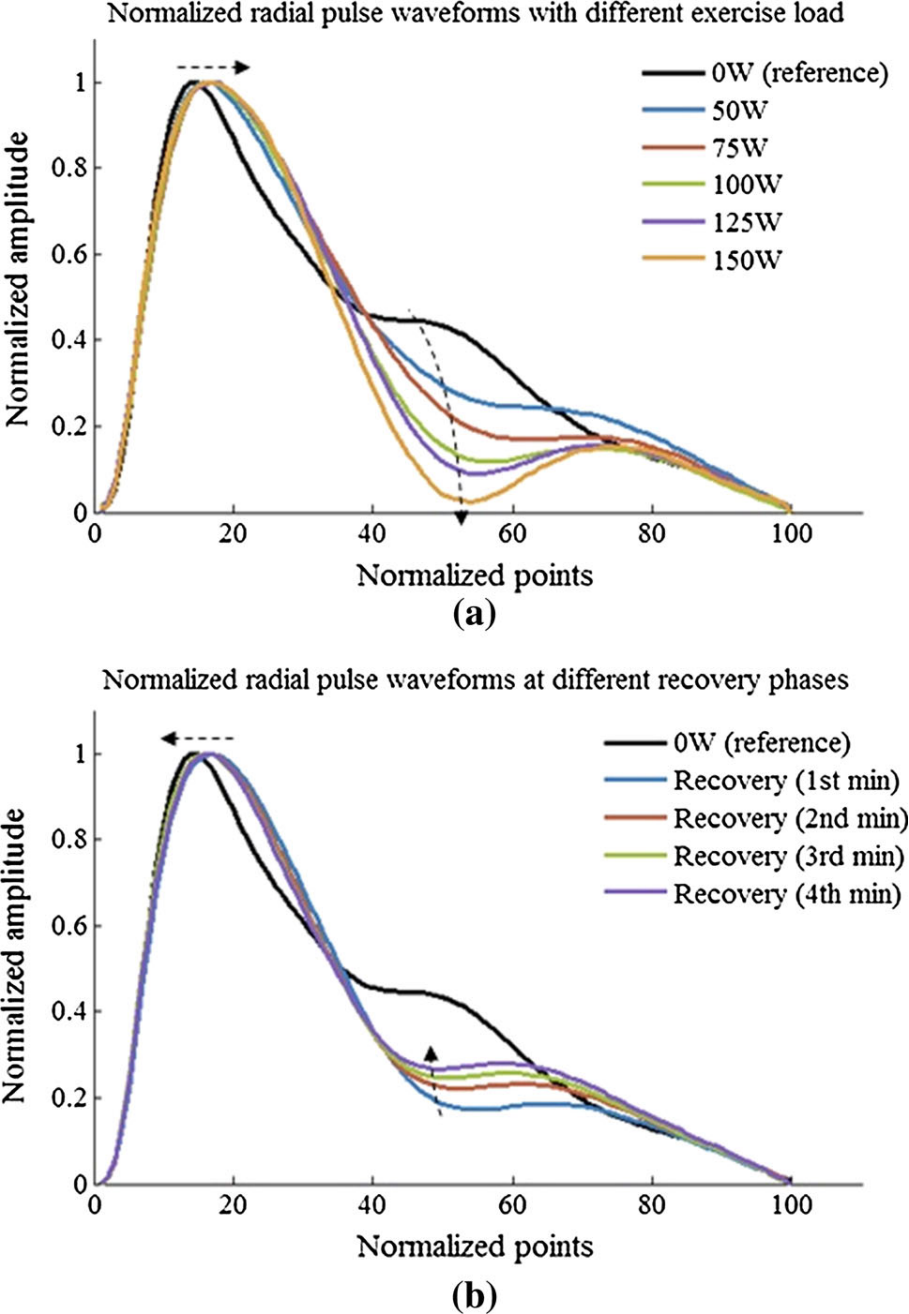
Normalized radial pulse waveforms averaged from 65 subjects studied. The *arrows* indicate the shift of the peak points and notch points during exercise and recovery

**Fig. 7:**
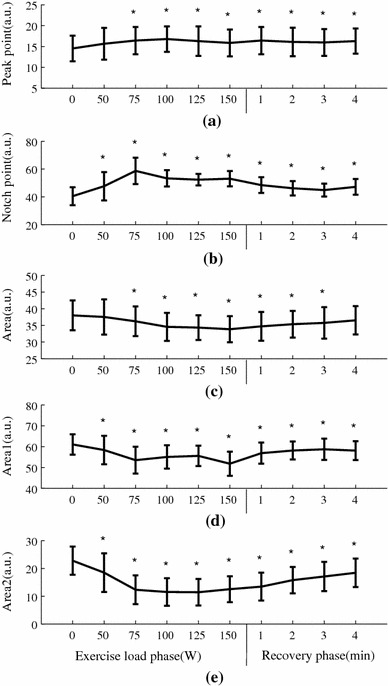
Normalized radial pulse waveform parameter (mean ± SD) changes of the 65 subjects studied. *P < 0.05 significantly different in comparison with the 0-W resting phase

Figure 6 shows the normalized radial pulse waveforms averaged from the 65 subjects studied, showing that the peak point position and notch point position were gradually moving to the right with increased exercise loads. During recovery, they were moving back to the normal position. More specifically, as shown in Fig. 7, the pulse peak point increased from 15 ± 3 at rest to 16 ± 3 at 150 W and then remained 16 ± 3 at the end of the 4-min recovery phase. The pulse peak points from the pulses with different exercise loads and recovery phase were significantly different when compared with the resting phase (all *P* < 0.05, except the 50-W exercise load phase). Correspondingly, the pulse notch point increased from 40 ± 6 at rest to 53 ± 6 at 150 W and then decreased to 47 ± 6 at the end of the 4-min recovery phase. All changes were significant (all *P* < 0.05).

In terms of the radial pulse Area, it decreased significantly during exercise from 38 ± 4 at rest to 34 ± 4 at 150 W and then increased to 37 ± 4 at the end of the 4-min recovery phase. There were significant differences in the pulse Area when comparing the exercise load and recovery phase with the resting phase except the 50-W exercise load phase and 4-min recovery phase (all *P* < 0.05). Correspondingly, pulse Area_1_ decreased from 61 ± 5 at rest to 52 ± 6 at 150 W and then increased to 58 ± 5 at the 4-min recovery phase. Pulse Area_2_ decreased from 23 ± 5 at rest to 13 ± 5 at 150 W and then increased back to 18 ± 5 at the 4-min recovery phase. All changes were significant (all *P* < 0.05).

### Effect of gender on arterial pulse waveform characteristics during exercise and recovery

The main difference was observed during the recovery period for the subjects studied. It has been found that the pulse Area and Area_1_ from male subjects were significantly smaller when compared with female subjects (all *P* < 0.05 for the four phases).

## Discussion and conclusion

The measurement of radial pulse is an effective method to monitor and evaluate the cardiovascular function. In this study, the waveform characteristics derived from the raw and normalized radial pulse waveform during exercise with different loads and during recovery have been quantitatively and comprehensively compared. To the best of our knowledge, it is the first comprehensive study to investigate the arterial pulse wave shape and its characteristic changes during exercise and recovery.

As expected, the SBP and pulse pressure gradually increased with heavier exercise loads. With increased exercise load, the body requires more blood to transport oxygen, and the ventricular systolic amplitude is increased to enlarge the blood-supply quantity, resulting in increased SBP [20]. However, our study showed that there were no significant differences in DBP between all the exercise loads or recovery phases and the resting phase, which agreed with some published studies [21]. During the exercise, an accelerated heart rate can lead to increased DBP, but skeletal muscle vasodilatation can lead to reduced DBP, resulting in unchanged DBP as a whole.

This study also showed that the pulse peak time *T*_p_ and dicrotic notch time *T*_n_ decreased with heavier exercise loads. It has been reported that the rising velocity of the pulse ascending curve is related to peripheral resistance, which is reduced because of the exercise muscle vasodilatation during exercise [22]. Therefore, the pulse peak time reduces with increasing exercise loads. Regarding the notch time, when the cardiac cycle has been shortened, the systolic and diastolic times are reduced at the same time, but the diastolic time has been reported to reduce more when compared with the systolic time [15], leading to the decreased pulse notch time *T*_n_ with increasing exercise loads.

The decreased normalized radial pulse Area has been observed in this study with increased exercise loads. This is consistent with the physiological changes. It has been reported that, during exercise, the increased cardiac ejection leads to a narrowed main wave and dicrotic wave [15]. Meanwhile, increased muscle metabolism causes muscle vasodilation and decreased muscle vascular resistance, resulting in lower dicrotic notch point [15]. The physiological changes in ejection function, peripheral resistance and blood vessel elasticity would ultimately reflect in the total pulse area changes as defined in this study. Our results agreed with some research reporting that vasodilation reduced the peripheral resistance during exercise, leading to a decreased total pulse Area with exercise [18, 23].

It has been reported that cardiovascular physiopathological characteristics during both systolic and diastolic phases are clinically useful for disease diagnosis [24]. During exercise, the cardiac systolic and diastolic phases would accordingly be shortened with increased heart rate, and the diastolic would be shortened more quickly. The excessive shortening of the diastolic period can cause ventricular filling and perfusion problems, leading to cardiac damage. In terms of the clinical significance of investigating the changes of Area_1_ and Area_2_, Plehn et al. [25] observed that the abnormal representation of the cardiac period was characterized by a prolongation of the left ventricular systole and an abnormal shortening of the left ventricular diastole in dilated cardiomyopathy patients. Additionally, the systolic-diastolic mismatch was accentuated during exercise and has the potential to impair the cardiac reserve in the patients with dilated cardiomyopathy by restricting ventricular filling and perfusion [25].

It has been accepted that the pulse waveform before the dicrotic notch point (pulse Area_1_) mainly reflects the systolic pulse wave characteristics, which are influenced by cardiac ejection function [26], and the arterial pulse waveform after the dicrotic notch point (pulse Area_2_) mainly reflects the diastolic pulse wave characteristics, which are affected by peripheral resistance and arterial compliance [27]. Specifically, it has been reported on radial arterial pulses that increased peripheral resistance is associated with a higher degree of dicrotic notch point and reflected pulse wave [27]. Therefore, the increased ejection function and decreased peripheral resistance induced by exercise [28, 29] could lead to reduced pulse Area_1_ and pulse Area_2_.

After exercise, subjects recovered to an unloading level with gradually decreased SBP, pulse pressure and heart rate. The pulse peak time *T*_p_, dicrotic notch time *T*_n_ and pulse Area, Area_1_ and Area_2_ increase gradually with the recovery time. However, it has been observed that, at the end of the 4-min recovery, not all of the waveform characteristics studied here returned to the baseline level, and the speed of recovery was also different. The quantification of their changes with a longer recovery time is therefore required in a future study. It was observed that the pulse Area and Area_1_ of male subjects were significantly smaller when compared with female subjects. A comprehensive comparison on more clinical parameters and with a specific study design is worthy of further investigation. Moreover, finger photoplethysmographic (PPG) pulses have often been recorded in cardiovascular research, and the underlying physiological mechanisms between arterial pressure pulses and finger PPG pulses could be different. Therefore, the changes of the PPG waveform characteristics and their association with arterial pulse waveform change during exercise and recovery would be worthy of further investigation.

In conclusion, this study quantitatively demonstrated significant changes of radial pulse waveform characteristics during different exercise loads and recovery phases.
